# Nigelladine A among Selected Compounds from *Nigella sativa* Exhibits Propitious Interaction with Omicron Variant of SARS-CoV-2: An In Silico Study

**DOI:** 10.1155/2023/9917306

**Published:** 2023-02-20

**Authors:** Md Mehedy Hasan Miraz, Md Afif Ullah, Abdullah Al Nayem, Brototi Chakrobortty, Sanjoy Deb, Anee Laskar, Nishita Umaya Tithi, Nilay Saha, Anita Rani Chowdhury, K. M. Khairul Alam, Tania Binte Wahed, Mohammad Khursheed Alam, Sukalyan Kumar Kundu

**Affiliations:** ^1^Department of Pharmacy, Jahangirnagar University, Savar, Dhaka 1342, Bangladesh; ^2^Department of Pharmacy, Jagannath University, Dhaka 1100, Bangladesh; ^3^Preventive Dentistry Department, Orthodontic Division, College of Dentistry, Jouf University, Sakaka 72345, Saudi Arabia; ^4^Department of Dental Research Cell, Saveetha Dental College and Hospitals, Saveetha Institute of Medical and Technical Sciences, Chennai, India; ^5^Department of Public Health, Faculty of Allied Health Sciences, Daffodil International University, Dhaka, Bangladesh

## Abstract

COVID-19 has been a threat to the entire world for more than two years since its outbreak in December 2019 in Wuhan city of China. SARS-CoV-2, the causative agent, had been reported to mutate over time exposing new variants. To date, no impeccable cure for the disease has been unveiled. This study outlines an extensive in silico approach to scrutinize certain phytochemical compounds of *Nigella sativa* (mainly the black cumin seeds) targeting the spike protein and the main protease (M^pro^) enzyme of the Omicron variant of SARS-CoV-2. The objective of this study is to investigate the extracted compounds with a view to developing a potential inhibitor against the concerned SARS-CoV-2 variant. The investigation contemplates drug-likeness analysis, molecular docking study, ADME and toxicity prediction, and molecular dynamics simulation which have been executed to elucidate different phytochemical and pharmacological properties of the tested compounds. Based on drug-likeness parameters, a total of 96 phytochemical compounds from *N. sativa* have been screened in the study. Interestingly, Nigelladine A among the compounds exhibited the highest docking score with both the targets with the same binding affinity which is −7.8 kcal/mol. However, dithymoquinone, kaempferol, Nigelladine B, Nigellidine, and Nigellidine sulphate showed mentionable docking scores. Molecular dynamics up to 100 nanoseconds were simulated under GROMOS96 43a1 force field for the protein-ligand complexes exhibiting the top-docking score. The root mean square deviations (RMSD), root mean square fluctuations (RMSF), radius of gyration (Rg), solvent accessible surface area (SASA), and the number of hydrogen bonds have been evaluated during the simulation. From the findings, the present study suggests that Nigelladine A showed the most promising results among the selected molecules. This framework, however, interprets only a group of computational analyses on selected phytochemicals. Further investigations are required to validate the compound as a promising drug against the selected variant of SARS-CoV-2.

## 1. Introduction

COVID-19 has been a trending issue since its outbreak in late December 2019 in the city of Wuhan, Hubei Province, China. The World Health Organization (WHO) announced this disease as a global pandemic on March 11, 2020 [1]. Due to the hasty spread of the virus, the socioeconomic condition of the entire world started to collapse. The World Health Organization (WHO) reported more than 617 million cases worldwide until September 30, 2022 [2]. In the course of time, more than 6.53 million deaths occurred due to COVID-19, which is massive from the usual perspective [2]. To date, an impeccable cure for this disease is still to be unveiled.

Severe acute respiratory syndrome coronavirus 2 (SARS-CoV-2), a ribonucleic acid (RNA) virus, is the causative agent of COVID-19 [3]. Undergoing extensive mutations, the virus generated a number of new variants, namely Omicron, Alpha, Beta, Delta, Gamma, and more [4]. WHO classified these variants into two types: variants of concern (VOCs) and variants of interest (VOIs) [4]. According to WHO, the Omicron B.1.1.529 variant was first reported to WHO on November 24, 2021, and WHO categorized it as a VOC for the first time on November 26, 2021 [4]. Since then, this variant has been ranked above other VOCs. This makes Omicron B.1.1.529 a more appropriate issue to scrutinize. Thus, this study aimed to investigate if there is any promising cure that can combat this variant.

Current findings demonstrate that mutations of SARS-CoV-2 variants are found to be more prevalent in the spike protein of the virus [5]. Hence, we have selected the spike protein as one of the targets in this study. This protein remains as a trimer on the surface of the viral envelope [6]. The receptor-binding domain (RBD) is possessed by the S1 domain and is particularly liable for binding the virus to the receptor [7, 8]. On the other hand, HR1 and HR2 are contained in the S2 domain, which is affiliated with viral fusion [8]. The RBD of the spike protein interacts with the host cells, admitting the receptor angiotensin-converting enzyme 2 (ACE2) [9]. Here, ACE2 itself also works as a receptor for the SARS-CoV-2 spike protein. By binding with ACE2, the virus facilitates endosome formation [9]. Consequently, it triggers viral fusion at a lower pH value [9]. This circumstance implies that, by intervening in the interaction between the spike protein and its receptor, the activity of the virus can be inhibited.

The main protease (M^pro^) in all variants of SARS-CoV-2 is an enzyme of SARS-CoV-2 that is broadly targeted by researchers. This enzyme plays an essential role in influencing viral replication and transcription [10]. The key function of this enzyme is to release functional polypeptides from each polyprotein through vast proteolytic processing [11]. The functions stimulate the replication of the virus, which is the key factor in its recurrence. M^pro^ is a homodimer that comprises two protomers each and incorporates three domains, namely domains I, II, and III [12]. Moreover, the human body does not possess a protein or enzyme nearly homologous to M^pro^. These cases note this enzyme as an ideal drug target to study. Interceding the process of this enzyme might lead to a solution to limit the recurrence of the virus. Thus, M^pro^ is included as one of the targets in this study.

Traditional medicines are playing a pivotal role in treating miscellaneous diseases, including a notable number of viral ones [13]. According to WHO, roughly 80% of the world's population relies on traditional medicines [14]. *Nigella sativa* (the black cumin seeds), belonging to the family Ranunculaceae, is one of the noteworthy plants with a significant medicinal profile [15]. Current literature states that *N. sativa* was confirmed to show antiasthmatic, anticancer, anti-inflammatory, antimicrobial, antioxidant, bronchodilator, hepato-protective, immunomodulator, renal protective, and many remarkable properties [16, 17]. More importantly, *N. sativa* showed promising activities against the SARS-CoV-2 Wuhan variant, which makes this plant a noteworthy herb to inspect [17]. Recent studies explored Nigellidine, nigellicine, nigellimine, thymol, *α*-hederin, thymoquinone, dithymoquinone, hederagenin, etc. compounds from this plant to inhibit selective targets of coronavirus [18]. Interestingly, the activities of this plant against Omicron variant were unexplored and missing in the current literature to date. Thus, *N. sativa* was picked in this study to scrutinize its activity against the targets of Omicron variant SARS-CoV-2.

Computer-aided drug design (CADD) via *in silico* methods is a convenient approach that accelerates the process of drug discovery and development [19]. In this computational study, we employed molecular docking, pharmacokinetic and pharmacodynamic property analyses, and molecular dynamics simulations to find the most suitable drug candidate. Molecular docking equipped us with data about the binding affinity, orientation, and type of interactions of each ligand with the respective target proteins. The pharmacokinetic profiles were acquired to investigate the data on the absorption, distribution, metabolism, and excretion (ADME) of the compounds that occur inside the body after drug administration. The toxicity study was carried out to get the LD50 values and toxicity classes of the individual ligands. Finally, molecular dynamics simulations were conducted to determine the stability and flexibility, along with certain properties, of the protein-ligand complexes.

In the present study, we approached a screening and 96 phytochemical compounds from *N. sativa* were sorted out based on the drug-likeness parameters. Through computational analyses, the molecules were tested in a wide spectrum. Hence, this framework interprets and presents some promising drug candidates from *N. sativa* against the SARS-CoV-2 Omicron B.1.1.529 variant.

## 2. Materials and Methods

### 2.1. Selection and Preparation of Ligands

On the basis of drug-likeness, a total of 96 phytochemical molecules from *Nigella sativa* were selected for this study. Lipinski's rule of five and Ghose's rules were considered while selecting the molecules [20, 21]. Only the molecules following both rules were picked for the study. The 3-dimensional (3D) conformers of the selected ligands were downloaded in SDF formats from the online databases of PubChem (https://pubchem.ncbi.nlm.nih.gov/) and IMPPAT 2.0 (Indian Medicinal Plants, Phytochemistry, and Therapeutics; https://cb.imsc.res.in/imppat/) [22, 23].

### 2.2. Retrieval and Preparation of Target Protein

The crystal structures of the spike protein (PDB ID: 7QNW) and M^pro^ (the main protease; PDB ID: 7TVX) of the SARS-CoV-2 Omicron B.1.1.529 variant were downloaded in PDB format from the database of the RCSB Protein Data Bank (https://www.rcsb.org/) [24, 25]. The resolutions of the downloaded spike protein and the M^pro^ were 2.40 Å and 2.094 Å, respectively. The protein structures were cleaned by removing undesired atoms and molecules (including ligands) using PyMOL version 2.5.2 software (Schrödinger, LLC) [26]. The receptor-binding domain (RBD) of the spike protein was isolated from the crystal structure, and the excessive chains of proteins were removed using PyMOL. This method was employed on M^pro^ also, and only one protein chain was kept. The energies of the selected protein chains of the spike protein and M^pro^ were minimized in Swiss-PdbViewer version 4.1.0 software using preset parameters [27]. The chains of the minimized proteins were saved in PDB formats for molecular docking and molecular dynamics simulations.

### 2.3. Molecular Docking

Molecular dockings on the selected ligands were performed against the target proteins using the CB-Dock2 server (https://cadd.labshare.cn/cb-dock2/php) [28]. The binding affinity (kcal/mol) for each protein-ligand complex as well as the noncovalent interactions and docking orientations were scrutinized by visualizing in the BIOVIA Discovery Studio 2021 Client version 21.1.0 software (Dassault Systèmes). The schematic illustrations of the protein-ligand docking complexes were retrieved in 2D and 3D forms from BIOVIA Discovery Studio.

### 2.4. ADME and Toxicity Prediction

The canonical SMILES of the ligands with the top-docking scores were copied from the PubChem and IMPPAT 2.0 databases and were inputted on the SwissADME server (https://www.swissadme.ch/) [29]. The ADME (absorption, distribution, metabolism, and excretion) data for each ligand were obtained from SwissADME. Subsequently, the toxicity profile of each ligand was predicted from the ProTox-II server (https://tox-new.charite.de/protox_II/) [30]. The physicochemical, pharmacokinetic, and pharmacodynamic properties of each ligand were noted from these two sources. The topological polar surface area (TPSA), lipophilicity (MLogP), water solubility (LogS), bioavailability score, blood-brain barrier (BBB) permeability, interaction with P-glycoprotein (P-gp), LD50 value, and toxicity class of each ligand were investigated during the ADME and toxicity prediction.

### 2.5. Molecular Dynamics Simulation

Molecular dynamics (MD) provides data on the stability and flexibility of protein-ligand complexes. The molecular dynamics were simulated using Groningen Machine for Chemical Simulations (GROMACS) software [31]. All simulation processes were carried out using the GROMOS96 43a1 force field. The MD simulation was conducted for up to 100 nanoseconds for each protein-ligand complex. At the onset, ligand topology files for each ligand were generated from the PRODRG server (https://davapc1.bioch.dundee.ac.uk/cgi-bin/prodrg/) by separating the ligands from the docked complexes retaining the same conformations and orientations [32]. During the simulation, the box was solvated with SPC water models, and the box type was set to a triclinic shape. To neutralize the system, 0.15 M NaCl salt was added to it. Structural optimization of 5000 steps was done by minimizing the energy of the system using the steepest descent algorithm. The simulation was carried out with equilibrium-type NVT and NPT. The temperature and the pressure of the system were maintained at 310 K and 1.0 bar, respectively, during the processes of simulation. The MD integration was done using the leap-frog method. From the results, the root mean square deviations (RMSD), root mean square fluctuations (RMSF), radius of gyration (Rg), solvent accessible surface area (SASA), and the number of hydrogen bonds in the protein-ligand complexes were scrutinized to get the most suitable molecule.

## 3. Results and Discussion

### 3.1. Molecular Docking and Noncovalent Interactions Analysis

**Figure 1:**
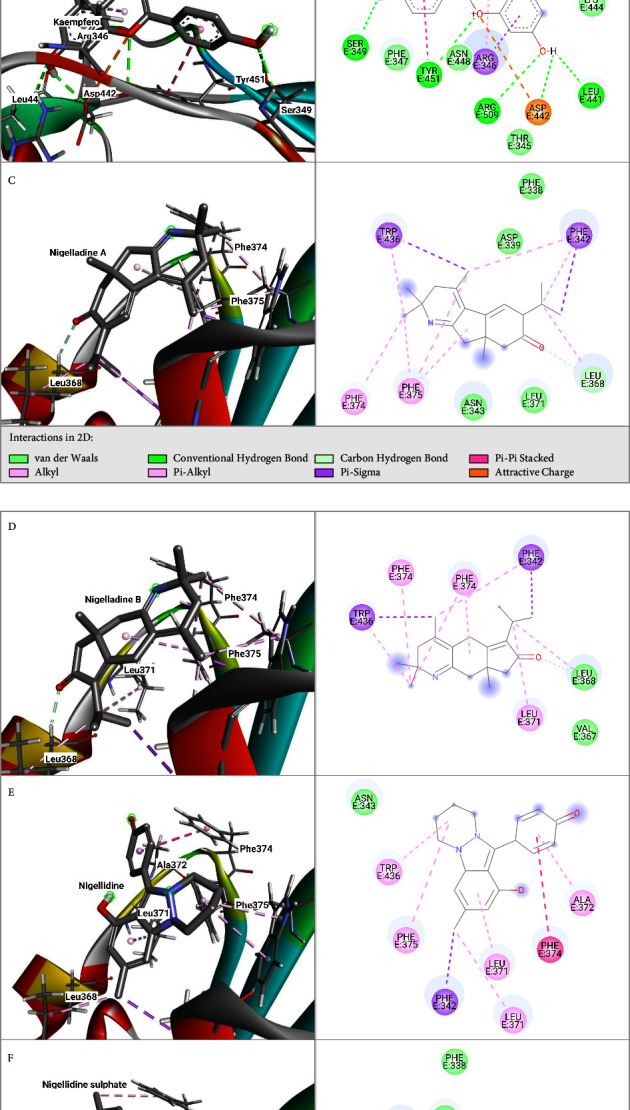
Docked conformations (3D and 2D) of spike protein with (A) dithymoquinone, (B) kaempferol, (C) Nigelladine A, (D) Nigelladine B, (E) Nigellidine, and (F) Nigellidine sulphate.

Nigelladine A showed the best docking scores against both target proteins, with a binding affinity of −7.8 kcal/mol in both cases. Interestingly, the top 6 molecules on the basis of binding affinity remain the same for both target proteins, only varying in binding affinities. Against the spike protein, the other molecules showing good binding affinity are kaempferol, Nigellidine, dithymoquinone, Nigellidine sulphate, and Nigelladine B, of which the binding affinities are −7.6, −7.5, −7.5, −7.4, and −7.3 kcal/mol, respectively. The binding affinities and the noncovalent interactions (hydrogen bonds and hydrophobic) of the top 6 molecules are presented in Table 1. The 3D and 2D docked conformations of the ligands with M^pro^ are shown in Figure 1.

Against the spike protein, Nigelladine A and Nigellidine sulphate each formed only one hydrogen bond with the residue LEU368 and showed four hydrophobic interactions with residues PHE342, PHE374, PHE375, and TRP436. Nigelladine B formed bonds with the same residues as Nigelladine A and Nigellidine sulphate had a hydrophobic interaction with LEU371. Kaempferol formed five hydrogen bonds and exhibited one hydrophobic interaction (Table 1). Nigellidine and dithymoquinone both did not form any hydrogen bonds. They showed seven and two hydrophobic interactions, respectively (Table 1).

**Figure 2:**
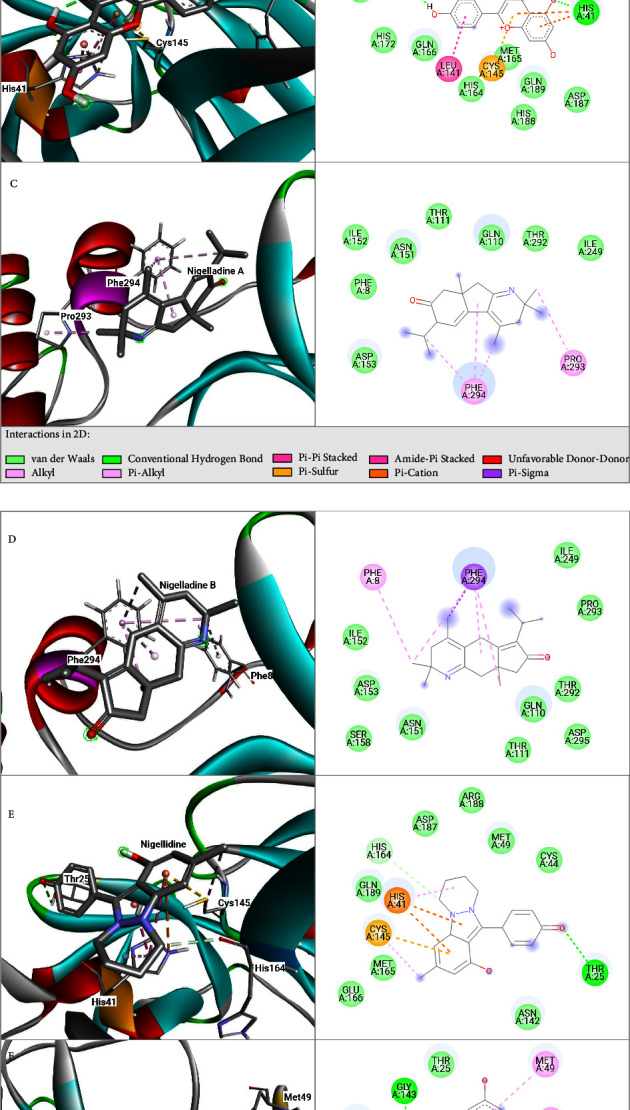
Docked conformations (3D and 2D) of M^pro^ with (A) dithymoquinone, (B) kaempferol, (C) Nigelladine A, (D) Nigelladine B, (E) Nigellidine, and (F) Nigellidine sulphate.

Against the main protease (M^pro^), the molecules alongside Nigelladine A exhibiting good affinities are Nigellidine sulphate, Nigellidine, kaempferol, dithymoquinone, and Nigelladine B with binding affinities of −7.8, −7.6, −7.2, −7.2, and −7.2 kcal/mol, respectively (Table 1). The 3D and 2D diagrams of docked conformations of the ligands with M^pro^ are shown in Figure 2. Nigelladine A showed no hydrogen bonds but hydrophobic bonds with residues PRO293 and PHE294. Nigellidine, Nigellidine sulphate, and dithymoquinone showed six, four, and two interactions, respectively, with half of the interactions being hydrogen bonds in each set of interactions. Nigelladine B showed two interactions, both of which are hydrophobic, and kaempferol showed three hydrogen bonds and one hydrophobic. The interacting residues are mentioned in Table 1.

### 3.2. ADME and Toxicity Analyses

The physicochemical, pharmacokinetic, and pharmacodynamic properties of the drug have been scrutinized using the data retrieved from SwissADME and Protox-II. ADME data of the molecules show detailed information, including molecular weight (MW), topological polar surface area (TPSA), lipophilicity (MLogP), water solubility (LogS), gastrointestinal absorption, bioavailability score, blood-brain barrier (BBB) permeability, and a complete profile of the molecules. These parameters indicate how suitably the molecules (entering the human body) will be absorbed, distributed, metabolized, and finally excreted. Moreover, the toxicity level of the molecules was taken into account, since no toxic molecule would be a suitable cure. The top 6 molecules based on molecular docking scores were considered for ADME and toxicity analyses. All molecules showed zero violations of Lipinski's rule of five and Ghose's rule.

Molecules with <90 Å^2^ TPSA tend to be permeable to the BBB, whereas those with >140 Å^2^ TPSA tend to be poorly permeable to the cell membrane [33, 34]. Nigelladine A and Nigelladine B exhibit the minimum TPSA, which is 29.43 Å^2^, whereas kaempferol occupied the highest value with an area of 111.13 Å^2^. Among the rest, three molecules showed TPSA of less than 90 Å^2^ (Table 2). This indicates that except for kaempferol and Nigellidine sulphate, other molecules were found to be BBB permeable. All the molecules having TPSAs <140 Å^2^ are able to permeate the cell membrane.

According to Lipinski's rule of five, oral drugs should have lipophilicity <5.0. The lipophilicity (MLogP) for kaempferol was noted as −0.03, which is the only negative value among the top 6 compounds. Nigelladine A and Nigelladine B displayed the highest value, which is 3.32. Nigellidine, Nigellidine sulphate, and dithymoquinone exhibited the MLogP values of 2.39, 2.17, and 1.74, respectively (Table 2). Nigelladine A, kaempferol, Nigellidine, Nigellidine sulphate, dithymoquinone, and Nigelladine B showed water solubility (LogS (ESOL)) values of −3.11, −3.31, −3.95, −4.51, −3.05, and −3.11, respectively. As per these, all molecules were properly water-soluble except Nigellidine sulphate (it was moderately soluble).

The gastrointestinal absorption of all molecules was high. Alongside, all molecules occupied the same bioavailability score, which is 0.55. However, only Nigellidine and Nigellidine sulphate were P-glycoprotein (P-gp) substrates. In terms of toxicity analysis, kaempferol and dithymoquinone took a safer place with lethal dose 50 (LD50) values of 3919 and 2300 mg/kg, respectively, belonging to toxicity class 5 (2000 < LD50 ≤ 5000) (Table 2). Nigelladine A and Nigelladine B showed the same and the lowest value, which is 900 mg/kg, addressing toxicity class 4 (300v< LD50 ≤ 2000).

**Figure 3:**
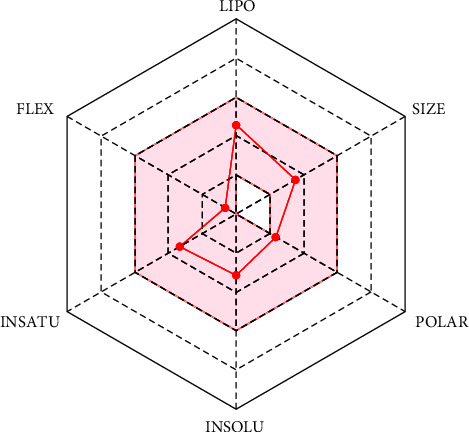
The bioavailability radar of Nigelladine A retrieved from SwissADME. The colored zone is the suitable physicochemical space for oral bioavailability. LIPO (lipophilicity): −0.7 < XLOGP3 < +5.0; SIZE: 150 g/mol < MV < 500 g/mol; POLAR (polarity): 20 Å^2^ < TPSA < 130 Å^2^; INSOLU (insolubility): −6 < Log S (ESOL) < 0; INSATU (insaturation): 0.25 < Fraction Csp3 < 1; FLEX (flexibility): 0 < num. rotatable bonds < 9.

To be administered orally, the drug candidate must follow certain criteria. Hence, the bioavailability radar of Nigelladine A depicts an overview of some major physicochemical criteria (Figure 3). Lipophilicity (in terms of XLogP3) between −0.7 and 5.0, size between 150 and 500 g/mol, TPSA between 20 and 130 Å^2^, LogS (ESOL) between −6 and 0, insaturation (fraction Csp3) between 0.25 and 1, and number of rotatable bonds between 0 and 9 are favorable for proper oral bioavailability. Nigelladine A remains in the favorable zone, occupying the suitable ranges for these criteria.

### 3.3. Molecular Dynamics Simulation Studies

In molecular dynamics simulation, the root mean square deviations (RMSD), root mean square fluctuations (RMSF), radius of gyration (Rg), solvent accessible surface area (SASA), and the number of hydrogen bonds in each protein-ligand complex were scrutinized to determine the most suitable drug candidate from the selected ligands.

#### 3.3.1. Root Mean Square Deviations (RMSD)

**Figure 4:**
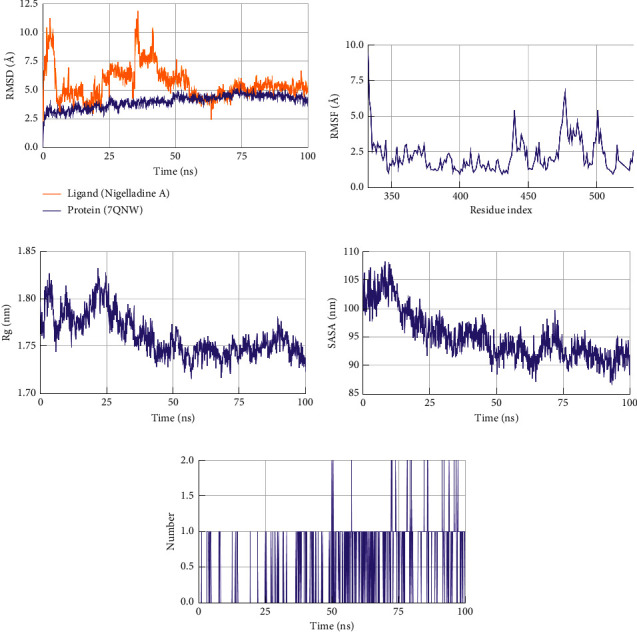
The (a) root mean square deviations (RMSD), (b) root mean square fluctuations (RMSF), (c) radius of gyration (Rg), (d) solvent accessible surface area (SASA), and (e) number of hydrogen bonds (h-bonds) plots for the protein-ligand complex of Nigelladine A and spike protein generated during MD simulation.

**Figure 5:**
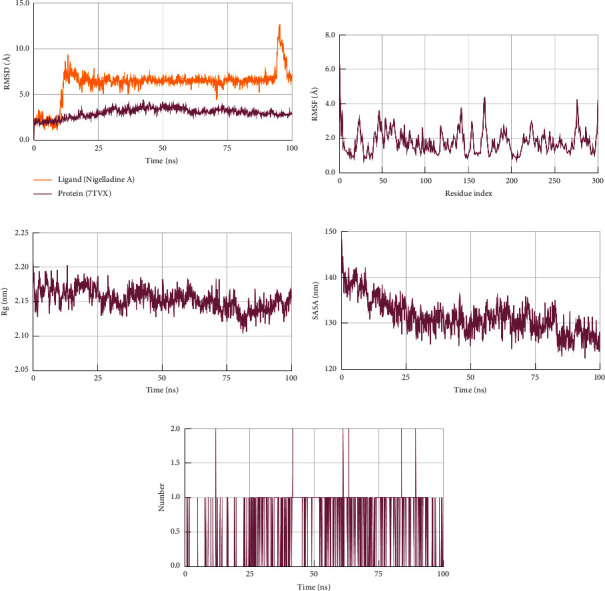
The (a) root mean square deviations (RMSD), (b) root mean square fluctuations (RMSF), (c) radius of gyration (Rg), (d) solvent accessible surface area (SASA), and (e) number of hydrogen bonds (h-bonds) plots for the protein-ligand complex of Nigelladine A and M^pro^ generated during MD simulation.

The root mean square deviations (RMSD) were evaluated to understand the stability of the protein-ligand complexes. A lower RMSD value is always favorable since it does not have any absolute rule. The average RMSD value for ligand Nigelladine A in the complex with spike protein was ∼5.6 Å ranging between ∼2.5–11.8 Å, and that of the backbone of spike protein was ∼4.0 Å ranging between ∼2.5–5.0 Å (Figure 4). The values for the ligand deviated unduly during the first half of the simulation. The latter half showed a smaller deviation. The average RMSD of Nigelladine A in the complex of the molecule with M^pro^ was ∼6.2 Å ranging between ∼1.2–12.6 Å (Figure 5). The outlying values in the graph peak near 95 ns in the simulation. The RMSD value of the backbone in the complex of Nigelladine A with M^pro^ ranges between ∼1.4–4.1 Å with an average of ∼3.0 Å. Nigelladine A depicts a more favorable graphical plot with M^pro^ than that with the spike protein.

#### 3.3.2. Root Mean Square Fluctuations (RMSF)

The root mean square fluctuations (RMSF) of the protein-ligand complexes were scrutinized to elucidate the flexibility of the protein structure (Figures 4 and 5). An RMSF value of 3.4 Å or below is considered ideal [35]. The RMSF value for the spike protein in the complex with Nigelladine A was observed between ∼0.93–9.45 Å with an average of 2.25 Å. In contrast, the residues of M^pro^ fluctuated between ∼0.72–6.28 Å with an average of 1.79 Å. Hence, both the target proteins are found to fluctuate within the ideal RMSF value. Hence, M^pro^ was observed to fluctuate less in comparison to the spike protein. The residues of the spike protein fluctuated below 6.00 Å except for residues 333, 334, and 477 (Figure 4). Moreover, all residues of M^pro^ except 1, 169, 276, and 300 fluctuated below 3.80 Å (Figure 5).

#### 3.3.3. Radius of Gyration (Rg)

The radius of gyration (Rg) indicates the radial distance to a certain point that would hold a moment of inertia identical to the actual mass distribution of the ligand if the ligand's total mass was concentrated. Thus, a lower Rg means tighter packing, whereas a higher Rg means looser packing of the protein. Based on the size and shape of the protein, it varies. The RBD of the spike protein and that of the M^pro^ carry 180 and 300 residues, respectively. These targets should have ideal values of 1.8 and 2.1 nm, respectively [36]. The Rg values of Nigelladine A in both complexes with proteins were analyzed to obtain the favorable one (Figures 4 and 5). The Rg of Nigelladine A in the complex with the spike protein demonstrated values within ∼1.72–0.83 nm with an average of ∼1.76 nm. The Rg in the other complex displayed a higher range of values. That of the same ligand in the complex with M^pro^ was between ∼2.10–2.21 nm, with an average of ∼2.15 nm.

#### 3.3.4. Solvent Accessible Surface Area (SASA)

The solvent accessible surface areas (SASA) of the ligand in the protein-ligand complexes describe the surface areas of the ligands that the solvent can access. Hence, there is no supreme value, as it depends on the size and shape of the complex. A lower SASA is always desired when comparing a group of molecules. The SASA values of Nigelladine A in both complexes were higher during the first 50 ns of simulation, and then, the values started to decrease gradually with a smaller range of fluctuations (Figures 4 and 5). Up to the full time of the simulation, the average SASA value of Nigelladine A in complex with the spike protein was recorded ∼95.03 nm^2^, whereas that of the same molecule in complex with M^pro^ was ∼131.37 nm^2^ (Figures 4 and 5). During the final 50 ns of the simulation, the SASA of Nigelladine A while in complex with the spike protein ranged between ∼86.60–99.76 nm^2^, showing ∼92.18 nm^2^ on average. That of Nigelladine A in complex with M^pro^ during the same interval of time was remarkably higher in value. That occupied an average of ∼129.55 nm^2^ ranging between ∼122.33–136.47 nm^2^.

#### 3.3.5. Hydrogen Bonds

Nigelladine A showed a maximum of 2 hydrogen bonds with both target proteins. The hydrogen bonds contribute favorably to the stability [37]. Thus, hydrogen bonds are always expected in terms of molecular dynamics studies. With the spike protein, the average number of hydrogen bonds forming during the simulation is ∼0.47 per timeframe, whereas that with M^pro^ is ∼0.56 per timeframe (Figures 4 and 5). There was a maximum of a single hydrogen bond with the spike protein recorded during the first half of the simulation. Several maxima were depicted in the graphical plots during the final half-time. In contrast, during the simulation with M^pro^, the maximal value was soon reached and repeated with different intervals of time.

## 4. Conclusion

This study aimed to investigate the promising molecules from *N. sativa* with a view to finding potential inhibitors against the Omicron variant of SARS-CoV-2. Among the tested compounds, Nigelladine A demonstrated the most promising results against both target proteins. In terms of binding affinity, Nigelladine A exhibited the top scores in both cases while docking with the two targets. Likewise, in the molecular dynamics simulation, this molecule retained pertinent results. However, this study only elucidates the *in silico* properties and profiles of the selected phytochemical compounds from *N. sativa*. Further experimental validation is required to confirm the activity of Nigelladine A as a potential inhibitor against the SARS-CoV-2 Omicron variant as well as for other variants. Hence, this study proposes Nigelladine A as a promising drug candidate showing favorable interactions against the studied targets of SARS-CoV-2.

## Figures and Tables

**Table 1:** The binding affinities and noncovalent (hydrogen bonds and hydrophobic) interactions of the ligands with top-docking scores against each protein.

Target protein	Ligand	Binding affinity (kcal/mol)	Noncovalent interactions	
			Hydrogen bonds	Hydrophobic
Spike protein	Dithymoquinone	−7.5	—	PHE342, LEU368
	Kaempferol	−7.6	SER349, LEU441, ASP442, TYR451, ARG509	ARG346
	Nigelladine A	−7.8	LEU368	PHE342, PHE374, PHE375, TRP436
	Nigelladine B	−7.3	LEU368	PHE342, LEU371, PHE374, PHE375, TRP436
	Nigellidine	−7.5	—	PHE342, LEU368, LEU371, ALA372, PHE374, PHE375, TRP436
	Nigellidine sulphate	−7.4	LEU368	PHE342, PHE374, PHE375, TRP436

M^pro^	Dithymoquinone	−7.2	THR292	PHE294
	Kaempferol	−7.2	HIS41, PHE140, ASN142	LEU141
	Nigelladine A	−7.8	—	PRO293, PHE294
	Nigelladine B	−7.2	—	PHE8, PHE294
	Nigellidine	−7.6	THR25, HIS164	HIS41, CYS145
	Nigellidine sulphate	−7.8	HIS41, ASN142, GLY143	CYS44, MET49, CYS145

**Table 2:** The physicochemical, pharmacokinetic, and pharmacodynamic properties of the molecules with the top 6 docking scores retrieved from SwissADME and Protox-II.

Parameters	Nigelladine A	Kaempferol	Nigellidine	Nigellidine sulphate	Dithymoquinone	Nigelladine B
MW (g/mol)	283.41	286.24	294.35	374.41	328.40	283.41
TPSA (Å^2^)	29.43	111.13	47.16	103.85	68.28	29.43
MLogP	3.32	−0.03	2.39	2.17	1.74	3.32
LogS (ESOL)	−3.11	−3.31	−3.95	−4.51	−3.05	−3.11
ESOL class	Soluble	Soluble	Soluble	Moderately soluble	Soluble	Soluble
GI absorption	High	High	High	High	High	High
Bioavailability score	0.55	0.55	0.55	0.55	0.55	0.55
BBB permeant	Yes	No	Yes	No	Yes	Yes
P-gp substrate	No	No	Yes	Yes	No	No
Lipinski vio	0	0	0	0	0	0
Ghose vio	0	0	0	0	0	0
LD50 (mg/kg)	900	3919	1000	1000	2300	900
Toxicity class	4	5	4	4	5	4

MW: molecular weight; TPSA: topological polar surface area; MLogP: lipophilicity; LogS (ESOL): water solubility; ESOL class: water solubility class; GI absorption: gastrointestinal absorption; bioavailability score: Abbott bioavailability score; BBB permeant: blood-brain barrier permeability; P-gp substrate: interaction with P-glycoprotein; Lipinski Vio: number of violations of Lipinski's rule of five; Ghose Vio: number of violations of Ghose's rule; LD50 (mg/kg): lethal dose 50; toxicity class: class based on LD50 value.

## Data Availability

All data are available within the manuscript.

## Supplementary Materials

Supplementary Materials The supplementary materials include the data of Lipinski's Rule of Five and Ghose's Rules Data and Docking scores.
