# Supplementary material for: Nigelladine A among Selected Compounds from Nigella sativa Exhibits Propitious Interaction with Omicron Variant of SARS-CoV-2: An In Silico Study
Source: Int J Clin Pract. 2023 Feb 20;2023:9917306. doi: 10.1155/2023/9917306 (PMC9970708; doi:10.1155/2023/9917306)
Supplement: Supplementary Materials — The supplementary materials include the data of Lipinski's Rule of Five and Ghose's Rules Data and Docking scores. [file 9917306.f1.docx]

**Supplementary Data**

**Nigelladine A among Selected Compounds from *Nigella sativa* Exhibits Propitious Interaction with Omicron Variant of SARS-CoV-2: An *In Silico* Study**

**Md Mehedy Hasan Miraz^1^, Md Afif Ullah^1^, Abdullah Al Nayem^1^, Brototi Chakrobortty^1^, Sanjoy Deb^1^, Anee Laskar^1^, Nishita Umaya Tithi^1^, Nilay Saha^1^, Anita Rani Chowdhury^1,2^, K.M. Khairul Alam^1^, Tania Binte Wahed^1^, Mohammad Khursheed Alam^3,4,5^ and Sukalyan Kumar Kundu^1^***

^1^ *Department of Pharmacy, Jahangirnagar University, Savar, Dhaka-1342, Bangladesh;*

^2^ *Department of Pharmacy, Jagannath University, Dhaka-1100, Bangladesh;*

^3^ *Preventive Dentistry Department, Orthodontic Division, College of Dentistry, Jouf University, Sakaka 72345, Saudi Arabia;*

*^4^ Department of Dental Research Cell, Saveetha Dental College and Hospitals, Saveetha Institute of Medical and Technical Sciences. Chennai, India;*

*^5^ Department of Public Health, Faculty of Allied Health Sciences, Daffodil lnternational University. Dhaka, Bangladesh.*

*** Corresponding Author**

Sukalyan Kumar Kundu

Department of Pharmacy, Jahangirnagar University

Savar, Dhaka, Bangladesh.

Email: skkbd415@juniv.edu

Phone: +8801731291468

**Contents**

Lipinski’s Rule of Five and Ghose’s Rules Data 3

Docking scores 8

**Lipinski’s Rule of Five and Ghose’s Rules Data on the 96 compounds from *Nigella sativa*:**

[Retrieved from SwissADME server ([www.swissadme.ch](http://www.swissadme.ch))]

Maximum 1 violation for Lipinski’s rule of 5, and NO violation of Ghose’s rule is allowed.

*[Sorted alphabetically.]*

| **S/L** | Chemical ID  (IMPPAT/ PubChem) | **Compound Name** | **Lipinski’s rule of 5 violation(s)** | **Ghose’s rule violation(s)** |
| --- | --- | --- | --- | --- |
| 1 | IMPHY011894 | (-)-7-Epi-alpha-selinene | 0 | 0 |
| 2 | IMPHY010609 | (-)-alpha-Himachalene | 0 | 0 |
| 3 | IMPHY003977 | (-)-beta-Bourbonene | 0 | 0 |
| 4 | IMPHY011957 | (+)-delta-Cadinene | 0 | 0 |
| 5 | IMPHY011793 | (+)-gamma-Cadinene | 0 | 0 |
| 6 | IMPHY004215 | (1R,2R,7R,8R)-2,6,6,9-tetramethyltricyclo[5.4.0.02,8]undec-9-ene | 0 | 0 |
| 7 | IMPHY015562 | (1R,4S,5R)-4-methoxy-4-methyl-1-propan-2-ylbicyclo[3.1.0]hexane | 0 | 0 |
| 8 | IMPHY004216 | (1S,2S,7S,8S)-2,6,6,9-tetramethyltricyclo[5.4.0.02,8]undec-9-ene | 0 | 0 |
| 9 | IMPHY012737 | (1S,4E,9S)-4,11,11-trimethyl-8-methylidenebicyclo[7.2.0]undec-4-ene | 0 | 0 |
| 10 | IMPHY011570 | (2Z,6E)-Farnesyl acetate | 0 | 0 |
| 11 | IMPHY000108 | (3S,4S)-4-ethenyl-4-methyl-3-prop-1-en-2-ylcyclohexene | 0 | 0 |
| 12 | IMPHY011631 | (E,Z)-farnesol | 0 | 0 |
| 13 | IMPHY014866 | 2-Cyclohexen-1-ol, 2-methyl-5-(1-methylethenyl)-, acetate, cis- | 0 | 0 |
| 14 | IMPHY001881 | 2-Tridecanone | 0 | 0 |
| 15 | IMPHY011521 | 2-Undecanone | 0 | 0 |
| 16 | IMPHY006243 | 2,5-Dimethoxy-p-cymene | 0 | 0 |
| 17 | IMPHY017689 | 2'-Hydroxy-5'-methoxyacetophenone | 0 | 0 |
| 18 | IMPHY015436 | 3-Buten-2-one, 4-(1,2,6,6-tetramethyl-2-cyclohexen-1-yl)- | 0 | 0 |
| 19 | IMPHY014907 | 6-Epi-beta-bisabolol | 0 | 0 |
| 20 | IMPHY008483 | 6,7-Dimethoxy-1-methylisoquinoline | 0 | 0 |
| 21 | IMPHY011589 | 7-epi-alpha-Eudesmol | 0 | 0 |
| 22 | IMPHY006709 | Acetyleugenol | 0 | 0 |
| 23 | IMPHY017038 | Alloisolongifolene | 0 | 0 |
| 24 | IMPHY013080 | alpha-Calacorene | 0 | 0 |
| 25 | IMPHY015123 | alpha-Copaene | 0 | 0 |
| 26 | IMPHY012279 | alpha-Curcumene | 0 | 0 |
| 27 | IMPHY011709 | alpha-Eudesmol | 0 | 0 |
| 28 | IMPHY005569 | alpha-Ionone | 0 | 0 |
| 29 | IMPHY017663 | alpha-Santalyl acetate | 0 | 0 |
| 30 | IMPHY011581 | alpha-Selinene | 0 | 0 |
| 31 | IMPHY011558 | Apiole | 0 | 0 |
| 32 | IMPHY014831 | beta-Caryophyllene | 0 | 0 |
| 33 | IMPHY010080 | beta-Elemene | 0 | 0 |
| 34 | IMPHY011542 | beta-Eudesmol | 0 | 0 |
| 35 | IMPHY009737 | beta-Longipinene | 0 | 0 |
| 36 | IMPHY014708 | beta-Selinene | 0 | 0 |
| 37 | IMPHY014942 | Bicyclo[2.2.1]heptan-2-ol, 1,7,7-trimethyl-, formate, (1R,2R,4R)-rel- | 0 | 0 |
| 38 | IMPHY014847 | Bornyl acetate | 0 | 0 |
| 39 | IMPHY014806 | Caswell No. 264AB | 0 | 0 |
| 40 | IMPHY011371 | cis-Chrysanthenyl acetate | 0 | 0 |
| 41 | IMPHY007421 | Citronellyl acetate | 0 | 0 |
| 42 | IMPHY008162 | Citronellyl butyrate | 0 | 0 |
| 43 | IMPHY014881 | Copaene | 0 | 0 |
| 44 | IMPHY009840 | Cyclosativene | 0 | 0 |
| 45 | IMPHY004209 | Davanone D | 0 | 0 |
| 46 | IMPHY003104 | Decanoic acid | 0 | 0 |
| 47 | IMPHY001144 | Dillapiol | 0 | 0 |
| 48 | IMPHY003467 | Dithymoquinone | 0 | 0 |
| 49 | IMPHY007204 | Dodecanal | 0 | 0 |
| 50 | IMPHY012638 | Epizonarene | 0 | 0 |
| 51 | IMPHY003536 | Eugenol | 0 | 0 |
| 52 | IMPHY011632 | Farnesol | 0 | 0 |
| 53 | IMPHY011777 | Farnesyl acetate | 0 | 0 |
| 54 | IMPHY012921 | gamma-Elemene | 0 | 0 |
| 55 | IMPHY005821 | gamma-Himachalene | 0 | 0 |
| 56 | IMPHY017035 | gamma-Thujaplicin | 0 | 0 |
| 57 | IMPHY001548 | Geranylacetone | 0 | 0 |
| 58 | IMPHY012738 | Isocaryophyllene | 0 | 0 |
| 59 | IMPHY008451 | Isolongifolene | 0 | 0 |
| 60 | CID_5280863 | Kaempferol | 0 | 0 |
| 61 | IMPHY003016 | Lauric acid | 0 | 0 |
| 62 | IMPHY012665 | Levomenol | 0 | 0 |
| 63 | IMPHY007067 | Linalyl acetate | 0 | 0 |
| 64 | IMPHY014513 | Longiborneol acetate | 0 | 0 |
| 65 | IMPHY005974 | Longicyclene | 0 | 0 |
| 66 | IMPHY004286 | Longifolene | 0 | 0 |
| 67 | IMPHY006177 | Methyl geranate | 0 | 0 |
| 68 | IMPHY006696 | Methyleugenol | 0 | 0 |
| 69 | IMPHY000060 | Myristic acid | 0 | 0 |
| 70 | CID_4276 | Myristicin | 0 | 0 |
| 71 | IMPHY003398 | Myristicin | 0 | 0 |
| 72 | IMPHY004634 | Myristoleic acid | 0 | 0 |
| 73 | IMPHY011648 | Neryl acetate | 0 | 0 |
| 74 | CID_12116700 | Nigeglanine | 0 | 0 |
| 75 | CID_86302612 | Nigelladine A | 0 | 0 |
| 76 | CID_86302611 | Nigelladine B | 0 | 0 |
| 77 | CID_86302610 | Nigelladine C | 0 | 0 |
| 78 | IMPHY001800 | Nigellicine | 0 | 0 |
| 79 | IMPHY000437 | Nigellidine | 0 | 0 |
| 80 | CID_139038581 | Nigellidine sulphate | 0 | 0 |
| 81 | IMPHY010712 | Nigellimine n-oxide | 0 | 0 |
| 82 | IMPHY013804 | Octyl isobutyrate | 0 | 0 |
| 83 | IMPHY000158 | Pyridoxine | 0 | 0 |
| 84 | CID_5280343 | Quercetin | 0 | 0 |
| 85 | IMPHY007840 | Spathulenol | 0 | 0 |
| 86 | IMPHY003537 | Tetradecanal | 0 | 0 |
| 87 | IMPHY000005 | Thiamine | 0 | 0 |
| 88 | IMPHY010815 | Thujopsene | 0 | 0 |
| 89 | IMPHY007606 | Thymohydroquinone | 0 | 0 |
| 90 | IMPHY001658 | Thymol methyl ether | 0 | 0 |
| 91 | IMPHY001218 | Thymoquinone | 0 | 0 |
| 92 | IMPHY016037 | trans-4-Methoxythujane | 0 | 0 |
| 93 | IMPHY016054 | trans-alpha-Bergamotene | 0 | 0 |
| 94 | IMPHY011354 | trans-Sabinene hydrate acetate | 0 | 0 |
| 95 | IMPHY009513 | Tridecanoic acid | 0 | 0 |
| 96 | IMPHY011745 | Zingiberene | 0 | 0 |

**Molecular Docking scores of the 96 compounds from *Nigella sativa* with the spike protein and M^pro^ of SARS-CoV-2 Omicron B.1.529:**

[Docking has been executed on CB-Dock2 server (<https://cadd.labshare.cn/cb-dock2>) via structure-based blind docking.]

*[Sorted alphabetically.]*

***N.B:*** *Chemical IDs start with “IMPHY” for molecules from IMPPAT server, and “CID_” for molecules from PubChem server.*

| **S/L** | **Chemical ID**  **(IMPPAT/ PubChem)** | **Compound Name** | **Binding affinity (kcal/mol)** | |
| --- | --- | --- | --- | --- |
|  |  |  | **Spike (7QNW)** | **M^pro^ (7TVX)** |
| 1 | IMPHY011894 | (-)-7-Epi-alpha-selinene | -6.8 | -6.1 |
| 2 | IMPHY010609 | (-)-alpha-Himachalene | -6.4 | -6.1 |
| 3 | IMPHY003977 | (-)-beta-Bourbonene | -6.1 | -6.1 |
| 4 | IMPHY011957 | (+)-delta-Cadinene | -6.8 | -6 |
| 5 | IMPHY011793 | (+)-gamma-Cadinene | -6.3 | -6 |
| 6 | IMPHY004215 | (1R,2R,7R,8R)-2,6,6,9-tetramethyltricyclo[5.4.0.02,8]undec-9-ene | -6.4 | -6.4 |
| 7 | IMPHY015562 | (1R,4S,5R)-4-methoxy-4-methyl-1-propan-2-ylbicyclo[3.1.0]hexane | -5.4 | -4.7 |
| 8 | IMPHY004216 | (1S,2S,7S,8S)-2,6,6,9-tetramethyltricyclo[5.4.0.02,8]undec-9-ene | -6.4 | -6.2 |
| 9 | IMPHY012737 | (1S,4E,9S)-4,11,11-trimethyl-8-methylidenebicyclo[7.2.0]undec-4-ene | -6.3 | -6.1 |
| 10 | IMPHY011570 | (2Z,6E)-Farnesyl acetate | -6.3 | -5.6 |
| 11 | IMPHY000108 | (3S,4S)-4-ethenyl-4-methyl-3-prop-1-en-2-ylcyclohexene | -5.4 | -5 |
| 12 | IMPHY011631 | (E,Z)-farnesol | -5.4 | -5.6 |
| 13 | IMPHY014866 | 2-Cyclohexen-1-ol, 2-methyl-5-(1-methylethenyl)-, acetate, cis- | -5.6 | -5.4 |
| 14 | IMPHY001881 | 2-Tridecanone | -5.1 | -4 |
| 15 | IMPHY011521 | 2-Undecanone | -4.8 | -4.1 |
| 16 | IMPHY006243 | 2,5-Dimethoxy-p-cymene | -5.3 | -4.9 |
| 17 | IMPHY017689 | 2'-Hydroxy-5'-methoxyacetophenone | -5.5 | -5.1 |
| 18 | IMPHY015436 | 3-Buten-2-one, 4-(1,2,6,6-tetramethyl-2-cyclohexen-1-yl)- | -5.7 | -5.2 |
| 19 | IMPHY014907 | 6-Epi-beta-bisabolol | -6.3 | -5.9 |
| 20 | IMPHY008483 | 6,7-Dimethoxy-1-methylisoquinoline | -5.6 | -5.4 |
| 21 | IMPHY011589 | 7-epi-alpha-Eudesmol | -6.6 | -6.7 |
| 22 | IMPHY006709 | Acetyleugenol | -5.8 | -5.9 |
| 23 | IMPHY017038 | Alloisolongifolene | -6 | -5.8 |
| 24 | IMPHY013080 | alpha-Calacorene | -6.6 | -6.1 |
| 25 | IMPHY015123 | alpha-Copaene | -6.4 | -5.9 |
| 26 | IMPHY012279 | alpha-Curcumene | -5.8 | -5.6 |
| 27 | IMPHY011709 | alpha-Eudesmol | -6.7 | -6.6 |
| 28 | IMPHY005569 | alpha-Ionone | -5.9 | -5.6 |
| 29 | IMPHY017663 | alpha-Santalyl acetate | -6.5 | -5.9 |
| 30 | IMPHY011581 | alpha-Selinene | -6.5 | -6.7 |
| 31 | IMPHY011558 | Apiole | -5.1 | -4.9 |
| 32 | IMPHY014831 | beta-Caryophyllene | -6.3 | -6.5 |
| 33 | IMPHY010080 | beta-Elemene | -6.2 | -5.6 |
| 34 | IMPHY011542 | beta-Eudesmol | -7.1 | -6.5 |
| 35 | IMPHY009737 | beta-Longipinene | -6.4 | -6.3 |
| 36 | IMPHY014708 | beta-Selinene | -6.8 | -6.4 |
| 37 | IMPHY014942 | Bicyclo[2.2.1]heptan-2-ol, 1,7,7-trimethyl-, formate, (1R,2R,4R)-rel- | -4.9 | -5.1 |
| 38 | IMPHY014847 | Bornyl acetate | -5.1 | -5.6 |
| 39 | IMPHY014806 | Caswell No. 264AB | -6.7 | -6 |
| 40 | IMPHY011371 | cis-Chrysanthenyl acetate | -5.5 | -5.4 |
| 41 | IMPHY007421 | Citronellyl acetate | -5.8 | -4.9 |
| 42 | IMPHY008162 | Citronellyl butyrate | -5.4 | -5.1 |
| 43 | IMPHY014881 | Copaene | -6.9 | -6.3 |
| 44 | IMPHY009840 | Cyclosativene | -6.4 | -6.1 |
| 45 | IMPHY004209 | Davanone D | -5.7 | -5.4 |
| 46 | IMPHY003104 | Decanoic acid | -5 | -4.6 |
| 47 | IMPHY001144 | Dillapiol | -5.4 | -5.3 |
| **48** | **IMPHY003467** | **Dithymoquinone** | **-7.5** | **-7.2** |
| 49 | IMPHY007204 | Dodecanal | -5.1 | -4.1 |
| 50 | IMPHY012638 | Epizonarene | -6.2 | -6.2 |
| 51 | IMPHY003536 | Eugenol | -5.9 | -5.1 |
| 52 | IMPHY011632 | Farnesol | -6.5 | -5.5 |
| 53 | IMPHY011777 | Farnesyl acetate | -6.2 | -5.6 |
| 54 | IMPHY012921 | gamma-Elemene | -6.2 | -5.7 |
| 55 | IMPHY005821 | gamma-Himachalene | -6.6 | -6.2 |
| 56 | IMPHY017035 | gamma-Thujaplicin | -5.6 | -5.5 |
| 57 | IMPHY001548 | Geranylacetone | -5.9 | -5.1 |
| 58 | IMPHY012738 | Isocaryophyllene | -6.2 | -5.7 |
| 59 | IMPHY008451 | Isolongifolene | -5.8 | -6.1 |
| **60** | **CID_5280863** | **Kaempferol** | **-7.6** | **-7.2** |
| 61 | IMPHY003016 | Lauric acid | -5.1 | -4.7 |
| 62 | IMPHY012665 | Levomenol | -6.3 | -6.3 |
| 63 | IMPHY007067 | Linalyl acetate | -5.2 | -5.1 |
| 64 | IMPHY014513 | Longiborneol acetate | -6.7 | -6.7 |
| 65 | IMPHY005974 | Longicyclene | -6.5 | -6.2 |
| 66 | IMPHY004286 | Longifolene | -6.4 | -6.1 |
| 67 | IMPHY006177 | Methyl geranate | -5.7 | -4.9 |
| 68 | IMPHY006696 | Methyleugenol | -5.8 | -4.9 |
| 69 | IMPHY000060 | Myristic acid | -5 | -5 |
| 70 | CID_4276 | Myristicin | -5.4 | -5.5 |
| 71 | IMPHY003398 | Myristicin | -5.4 | -5.2 |
| 72 | IMPHY004634 | Myristoleic acid | -5.7 | -4.7 |
| 73 | IMPHY011648 | Neryl acetate | -5.6 | -5.3 |
| 74 | CID_12116700 | Nigeglanine | -6 | -6.3 |
| **75** | **CID_86302612** | **Nigelladine A** | **-7.8** | **-7.8** |
| **76** | **CID_86302611** | **Nigelladine B** | **-7.3** | **-7.2** |
| 77 | CID_86302610 | Nigelladine C | -6.3 | -7.1 |
| 78 | IMPHY001800 | Nigellicine | -6.1 | -7 |
| **79** | **IMPHY000437** | **Nigellidine** | **-7.5** | **-7.6** |
| **80** | **CID_139038581** | **Nigellidine sulphate** | **-7.4** | **-7.8** |
| 81 | IMPHY010712 | Nigellimine n-oxide | -5.3 | -5.5 |
| 82 | IMPHY013804 | Octyl isobutyrate | -4.8 | -3.9 |
| 83 | IMPHY000158 | Pyridoxine | -4.6 | -4.9 |
| 84 | CID_5280343 | Quercetin | -7 | -7.1 |
| 85 | IMPHY007840 | Spathulenol | -6.1 | -6.4 |
| 86 | IMPHY003537 | Tetradecanal | -4.9 | -4.2 |
| 87 | IMPHY000005 | Thiamine | -5.4 | -5.2 |
| 88 | IMPHY010815 | Thujopsene | -6.2 | -6.1 |
| 89 | IMPHY007606 | Thymohydroquinone | -5.8 | -5.4 |
| 90 | IMPHY001658 | Thymol methyl ether | -5.4 | -4.9 |
| 91 | IMPHY001218 | Thymoquinone | -5.6 | -5.3 |
| 92 | IMPHY016037 | trans-4-Methoxythujane | -5.2 | -5 |
| 93 | IMPHY016054 | trans-alpha-Bergamotene | -6.4 | -5.7 |
| 94 | IMPHY011354 | trans-Sabinene hydrate acetate | -5.7 | -5.4 |
| 95 | IMPHY009513 | Tridecanoic acid | -4.6 | -4.7 |
| 96 | IMPHY011745 | Zingiberene | -6.4 | -5.3 |
